# Risk factors for parastomal hernia after abdominoperineal resection of rectal cancer

**DOI:** 10.3389/fonc.2024.1470113

**Published:** 2024-10-14

**Authors:** Lele Zhu, Shun Li, Feitong Wang

**Affiliations:** ^1^ Department of General Surgery, The Affiliated Hospital of Xuzhou Medical University, Xuzhou, Jiangsu, China; ^2^ Department of Neurology, University of Pittsburgh School of Medicine, Pittsburgh, PA, United States; ^3^ Cancer Institute, Xuzhou Medical University, Xuzhou, Jiangsu, China

**Keywords:** rectal cancer, parastomal hernia, miles operation, risk factors, abdominoperineal resection of rectal cancer

## Abstract

**Purpose:**

To investigate risk factors associated with the formation of parastomal hernia after Miles operation, and to provide scientific evidence for the prevention and treatment of parastomal hernia.

**Methods:**

Clinical data from 205 patients with rectal cancer undergoing Miles operation in the Department of General Surgery, Affiliated Hospital of Xuzhou Medical University between May 2016 and May 2021 were analyzed retrospectively. Fourteen potential factors were selected and analyzed by single factor analysis and two element logistic regression analysis for their potential relationship to incidence of parastomal hernia.

**Results:**

49 cases of parastomal hernia occurred among 194 patients during follow-up (incidence 25.26%). Univariate analysis showed that age, thickness of subcutaneous abdominal fat, BMI, and stoma pathway were related to the formation of post-surgical parastomal hernia (P < 0.05). Two element logistic regression analysis showed that advanced age, thickness of subcutaneous abdominal fat, BMI > 25 kg/m^2^, and transperitoneal surgical approach were independent risk factors for the formation of parastomal hernia after Miles operation (P < 0.05).

**Conclusion:**

Advanced age, thickness of subcutaneous abdominal fat, BMI > 25 kg/m^2^, and transperitoneal surgical approach are independent risk factors for the formation of parastomal hernia after Miles.

## Introduction

Parastomal hernia is a type of incisional hernia of the abdominal wall located adjacent to a stoma. It is an abnormal protrusion of the contents of the abdominal cavity through an abdominal wall defect created during placement of a colostomy, ileostomy, or ileal conduit stoma. Parastomal hernia is one of the most common long-term complications after Miles operation for rectal cancer (1). Most early patients are asymptomatic, or have mild abdominal discomfort. Although it is unable to solve the problem radically, surgical treatment is necessitated for some patients with severe parastomal hernia due to unbearable abdominal pain, side leakage of a stoma device, skin irritation around the stoma, and cosmetic problems caused by abdominal bulging (2–4). In addition to the high incidence of complications, the recurrence rate can still be as high as 30-76% even with mesh repair (5–7). Therefore, it is of great clinical significance to determine risk factors for parastomal hernia to prevent hernia formation.

Parastomal hernia is the result of the interaction of multiple factors. Although obesity, old age, glucocorticoid use, incision infection, and other factors have been reported as potential risk factors for parastomal hernia of patients in Europe and North America (8–10), these factors have not yet been fully validated. Moreover, there are few clinical studies on risk factors for parastomal hernia among Chinese population. Herein, we retrospectively analyzed the clinical data of 205 patients who underwent Miles operation for rectal cancer from May 2016 to May 2021 in a single institute, and explored the influencing factors of parastomal hernia in order to provide a scientific basis for its prevention and treatment.

## Materials and methods

### Patient cohort

Patients with rectal cancer who underwent Miles operation in the Affiliated Hospital of Xuzhou Medical University (China) between May 2016 and May 2021 were followed up for over 2 years and identified as potential candidates. They have complete clinical data for follow-up, and patients and their families cooperate with the follow-up. The endpoint of follow-up for deceased patients is the day of death, while non deceased patients must undergo follow-up for more than 2 years. Patients undergoing follow-up need to go to the hospital for an abdominal CT scan every three months.

### Diagnosis of parastomal hernia

This study used the definition and classification of parastomal hernia recommended by the European Hernia Society (EHS, 2013) to diagnose whether parastomal hernia occurred after Miles operation for rectal cancer (11) (**Table 1**). The definition is as follows: “Parastomal hernia is an abnormal protuberance of the contents of the abdominal cavity through the abdominal wall defect created during placement of a colostomy, ileostomy, or ileal conduit stoma. It should be distinguished from local stoma problems without a hernia sac, such as a mucosal prolapse or a Siphon loop, which is a subcutaneous folding of the excess bowel length at the stoma.” (11). Abdominal CT was performed within 2 years after operation, which is the diagnostic basis for the occurrence and clinical evaluation of parastomal hernia.

**Table 1:** EHS grid for classification of parastomal hernias.

Type	Defect size (cm)	cIH#
I	≤ 5	no
II	≤ 5	yes
III	> 5	no
IV	> 5	yes

# cIH, concomitant incisional hernias.

### Study design

Patients with parastomal hernia after Miles operation were selected as the case group, and patients without parastomal hernia after operation were set as the control group. Fourteen potential factors affecting the occurrence of parastomal hernia were selected, including gender, age, body mass index (BMI), albumin, previous abdominal surgery, preoperative chemoradiotherapy, hypertension, diabetes mellitus, chronic obstructive pulmonary disease, thickness of subcutaneous abdominal fat, stoma position (transrectus/lateral pararectus), stoma pathway (transperitoneal/extraperitoneal), type of approach (open/laparoscopic), and incision liquefaction and/or infection. Among them, the measurement of thickness of subcutaneous abdominal fat is done through preoperative abdominal CT.

### Statistical analysis

SPSS 21.0 software was used to perform single factor analysis (independent sample t-test, χ^2^ test, or Fisher exact probability method) and two element logistic regression analysis on 14 candidate risk factors that may affect the occurrence of parastomal hernia, in order to identify independent risk factors for parastomal hernia after Miles operation. P < 0.05 was considered statistically significant.

## Results

### Patient characteristics

**Figure 1:**
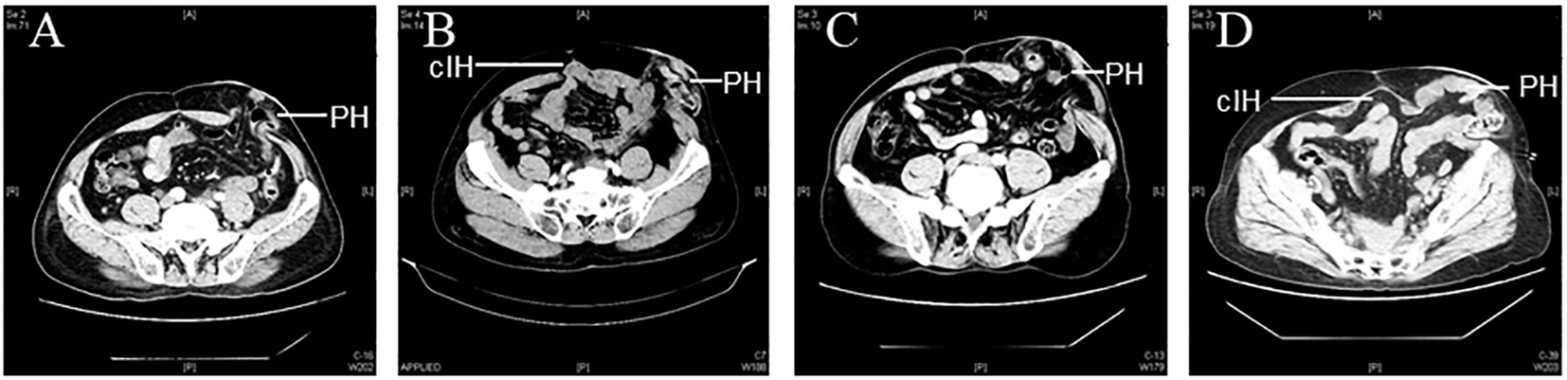
Representative CT images of parastomal hernia. (PH: parastomal hernias; cIH: concomitant incisional hernias) **(A)** Type I: PH≤ 5 cm without cIH; **(B)** Type II: PH ≤5 cm with cIH; **(C)** Type III: PH>5 cm without cIH; **(D)** Type IV: PH>5 cm with cIH.

A total of 205 patients with rectal cancer who underwent Miles operation were collected in this study. Among these, 11 cases were lost to follow-up and were not included in the analysis. The median follow-up period for the remaining 194 patients was 33.7 months (range, 1-76.9 months), and the abdominal CT was reviewed for all 194 patients after operation to diagnose the occurrence of parastomal hernia. Among them, 49 cases developed parastomal hernia (25.26% incidence), with 23 cases of type I, 5 cases of type II, 15 cases of type III, and 6 cases of type IV (**Figure 1**). There were 95 males (49%) and 99 females (51%), with 21-85 (60.99 ± 11.22) age range. Additional demographic information is shown in **Table 2**.

**Table 2:** Risk factors for parastomal hernia (univariate analysis).

variable	parastomal hernia (+)	parastomal hernia (-)	χ^2^ or t	P
age (y)	64.08 ± 10.37	59.94 ± 11.33	2.256	0.025^a^
albumin (g/L)	43.17 ± 3.54	41.81 ± 4.58	1.890	0.060^a^
thickness of subcutaneous abdominal fat (mm)	23.45 ± 9.23	18.52 ± 8.36	3.476	0.001^a^
sex				
Male	22	73	0.435	0.620^b^
Female	27	72		
BMI > 25 kg/m^2^				
Yes	24	37	9.352	0.004^b^
No	25	108
previous abdominal surgery				
Yes	4	14	0.001	1.000^c^
No	45	131
preoperative chemoradiotherapy				
Yes	2	3	0.061	0.602^c^
No	47	142
hypertension				
Yes	11	19	2.447	0.168^b^
No	38	126
diabetes mellitus				
Yes	5	11	0.076	0.556^c^
No	44	134
COPD				
Yes	2	4	< 0.001	0.644^c^
No	47	141
stoma pathway				
transperitoneal	47	105	11.928	< 0.001^b^
extraperitoneal	2	40
stoma position				
transrectus	16	47	0.001	1.000^b^
lateral pararectus	33	98
type of approach				
open	17	48	0.042	0.862^b^
laparoscopic	32	97
incision liquefaction and/or infection				
Yes	5	7	1.015	0.183^c^
No	44	138

a, independent sample t-test; b, χ^2^ test; c, corrected χ^2^ test.

### Single factor analysis

Univariate analysis was performed with 14 clinicopathological factors. **Table 2** displays the association between patient characteristics and parastomal hernia development. Upon analysis, the age of patients with parastomal hernia was significantly higher than that of patients without parastomal hernia (64.08 ± 10.37y vs. 59.94 ± 11.33y, p=0.025). Also, the thickness of subcutaneous fat in abdomen was greater in the group with parastomal hernia than that without (23.45 ± 9.23mm vs. 18.52 ± 8.36mm, p=0.001). The incidence of parastomal hernia in obese group (BMI ≥ 25 kg/m2) was higher than that in nonobese group (BMI < 25 kg/m2) ([24/62] 39.3% vs. [25/133] 18.8%, p=0.004). In addition, the stoma pathway affected the hernia formation significantly, with less incidence of extraperitoneal stoma than that of transperitoneal stoma ([2/42] 4.8% vs. [47/152] 30.9%, p<0.001).

### Two element logistic regression analysis

Further two element logistic regression analysis showed the similar patient characteristics with above single factor analysis: advanced age, thicker subcutaneous abdominal fat, BMI ≥ 25 kg/m2, and transperitoneal stoma pathway were independent risk factors for the formation of parastomal hernia after Miles operation (P < 0.05). See **Table 3**.

**Table 3:** Independent significant factors for parastomal hernia (two element logistic regression analysis).

variable	B	S.E.	OR	95% CI	P
age	0.045	0.019	1.046	1.009-1.085	0.015
subcutaneous abdominal fat (mm)	0.059	0.024	1.061	1.013-1.111	0.012
BMI > 25 kg/m^2^	-0.861	0.394	0.423	0.195-0.916	0.029
stoma pathway	2.705	0.790	14.951	3.180-70.280	0.001
constant	-6.957	1.628			

## Discussion

Rectal cancer is one of the most common malignant tumors of the digestive tract in China. In recent years, the incidence of rectal cancer has increased according to the changes in lifestyle, dietary structure, and population aging. As a result of in-depth study of the pathological and physiological characteristics of rectal cancer and the development of surgical instruments, the rate of anus-preserving surgery for low rectal cancer has increased from 40% to about 70% (12). However, for some patients with ultra-low rectal cancer, such as old age, obesity or pelvic narrowness, excessive pursuit of anus preservation only increases the probability of poor anal defecation control, anastomotic leakage and anastomotic stenosis after operation. Therefore, Miles operation for rectal cancer is still an irreplaceable operation in clinic.

Parastomal hernia is one of the most common long-term complications after Miles operation for rectal cancer (13, 14). The incidence of parastomal hernia is high, and most often occurs within 2 years after stoma operation (10, 15, 16). However, the incidence of parastomal hernia varies greatly, which may be related to the lack of a universally accepted definition and classification, as well as differing diagnostic methods and follow-up times (13, 15, 17). In this study all 194 patients were from one institute, diagnosed with CT scan, and followed up for more than 2 years. The incidence of parastomal hernia was 25.26%, within the worldwide range of 3% - 48% (18).

We investigated the risk factors associated with the formation of parastomal hernia after Miles operation in Chinese population. Our results showed that advanced age, thickness of subcutaneous abdominal fat, BMI ≥ 25 kg/m^2^, and transperitoneal surgical approach were independent risk factors for the formation of parastomal hernia after Miles operation. Increasing age was associated with greater probability of parastomal hernia, which is consistent with most research results worldwide (9, 19, 20). This may be due to the thinning of abdominal wall muscles, decreased strength, and increased subcutaneous fat thickness with increasing age. Weak abdominal wall muscles are insufficient to protect the defect caused by stoma, such that the contents of the abdominal cavity protrude from the skin surface through the defect in the abdominal wall. In addition, the capacity for tissue repair and nutritional status of elderly patients after operation are poor. These patients often have comorbid chronic diseases that increase abdominal pressure, which may contribute to increased incidence of parastomal hernia after Miles operation for rectal cancer.

Obesity is considered to be a risk factor for hernia including incisional hernia and parastomal hernia (9, 19, 21). It has been confirmed that obesity is an important factor in parastomal hernia in Japanese and Korean population (9, 19). The results of this study showed that overweight patients were more likely to develop parastomal hernia than non-overweight patients (39.3% vs. 18.8%). It occurs due to the increased thickness of abdominal subcutaneous fat and abdominal pressure, the weakness of abdominal muscles, as well as the increased risk of incision liquefaction or infection in patients with obesity. Abdominal contents protrude more easily through abdominal defects. Obese patients, especially those with abdominal obesity, have difficulty in orienting the stoma before operation (20). Our study incorporated thickness of subcutaneous abdominal fat as a new index to explore its relationship with parastomal hernia after Miles operation for rectal cancer. This is based on Kanehisa’s viewpoint (22) that thickness of subcutaneous fat in the abdomen can better reflect abdominal circumference, which is well accepted in the worldwide as an indicator of abdominal obesity. Abdominal obesity can better predict the risk of certain diseases than BMI, including type 2 diabetes, cerebrovascular accident, ischemic heart disease, etc (23). In our study, thickness of subcutaneous abdominal fat was associated with parastomal hernia after Miles and is an independent risk factor (OR, 1.061, 95% CI, 1.013-1.111, p=0.012<0.05).

Extraperitoneal stoma was first proposed by Amussant then applied in the clinic (24). This approach was shown by Goligher (25), Lian (26), and Hino (27) to significantly reduce the incidence of parastomal hernia. The incidence of parastomal hernia through extraperitoneal stoma (4.8%) was significantly lower than that through peritoneum (30.9%) in our study. It indicated that intraperitoneal hernia could be avoided by extraperitoneal drainage of the stoma intestine and mesentery and by changing the stoma intestine as an extraperitoneal organ. In addition to ensure the integrity of the peritoneum, extraperitoneal stoma effectively cushions the pressure of the intestinal canal on the anterior wall of the abdomen, conferring uniform stress on the peritoneum and the periostomy. The stoma intestinal canal is present in the extraperitoneal tunnel. The anterior abdominal muscles form a structure similar to the anterior wall of the inguinal canal, which strengthens the front of the tunnel and reduces the risk of hernia. When the intestinal canal passes through the tunnel, it forms a close adhesion between the peritoneum and rectus abdominis without leaving any space, thereby reducing the risk of parastomal hernia and prolapse of the stoma (28).

The results of this study show that advanced age, thickness of subcutaneous abdominal fat, BMI ≥ 25 kg/m^2^, and transperitoneal surgical approach are independent risk factors for the formation of parastomal hernia after Miles operation. These observations will guide in selecting the most appropriate approach for generating stoma for patients with multiple risk factors during Miles operation, or to pre-install patches at the same time as stoma formation, in order to prevent the occurrence of parastomal hernia and improve patient quality of life. Many studies have shown that prophylactic placement of patches after MILES surgery for rectal cancer can reduce the incidence of parastomal hernia without increasing postoperative complications (29, 30). The above content is the research results of our center. This study is a retrospective study of a patient cohort from a single institution. In the future, a multicenter retrospective study will be conducted in conjunction with other large hospitals in China to further validate the relevant research results.

## Data Availability

The original contributions presented in the study are included in the article/supplementary material. Further inquiries can be directed to the corresponding authors.
